# Wireless Fingerprinting Uncertainty Prediction Based on Machine Learning

**DOI:** 10.3390/s19020324

**Published:** 2019-01-15

**Authors:** You Li, Zhouzheng Gao, Zhe He, Yuan Zhuang, Ahmed Radi, Ruizhi Chen, Naser El-Sheimy

**Affiliations:** 1 Department of Geomatics Engineering, University of Calgary, 2500 University Dr NW, Calgary, AB T2N 1N4, Canada; liyou331@gmail.com (Y.L.); hezhe310@gmail.com (Z.H.); ahmed.elboraee@ucalgary.ca (A.R.); elsheimy@ucalgary.ca (N.E.-S.); 2 School of Land Science and Technology, China University of Geosciences (Beijing), 29 Xueyuan Road, Beijing 100083, China; 3 German Research Centre for Geosciences (GFZ), Telegrafenberg, 14473 Potsdam, Germany; 4 State Key Laboratory of Surveying, Mapping and Remote Sensing, Wuhan University, 129 Luoyu Road, Wuhan 430079, China; zhy.0908@gmail.com (Y.Z.); ruizhi.chen@whu.edu.cn (R.C.)

**Keywords:** indoor localization, fingerprinting, machine learning, neural network, received signal strength, Kalman filter, inertial navigation

## Abstract

Although wireless fingerprinting has been well researched and widely used for indoor localization, its performance is difficult to quantify. Therefore, when wireless fingerprinting solutions are used as location updates in multi-sensor integration, it is challenging to set their weight accurately. To alleviate this issue, this paper focuses on predicting wireless fingerprinting location uncertainty by given received signal strength (RSS) measurements through the use of machine learning (ML). Two ML methods are used, including an artificial neural network (ANN)-based approach and a Gaussian distribution (GD)-based method. The predicted location uncertainty is evaluated and further used to set the measurement noises in the dead-reckoning/wireless fingerprinting integrated localization extended Kalman filter (EKF). Indoor walking test results indicated the possibility of predicting the wireless fingerprinting uncertainty through ANN the effectiveness of setting measurement noises adaptively in the integrated localization EKF.

## 1. Introduction

The location information has become essential for the location-based service (LBS) applications. Global navigation satellite systems (GNSS, e.g., Global positioning systems (GPS), BeiDou navigation satellite systems (BDS), GLONASS and Galileo) have achieved great success and commercialization during the past decades for outdoor applications. However, their performances may be degraded in indoor environments. To fill the gap for indoor mass-market localization, indoor localization with consumer portable devices has been researched.

Among various indoor localization techniques, wireless localization has been widely used due to the promotion of access points (APs, e.g., based stations, beacons, and gateways) for wireless technologies such as low-power wide-area network (LPWAN), cellular network, wireless local area network (WiFi), Bluetooth low energy (BLE), ZigBee, ultra-wide-band (UWB), and radio frequency identification (RFID). Among various wireless measurements (e.g., time-of-arrival (ToA) [1], angle-of-arrival (AoA) [2], and received signal strength (RSS) [3]), RSS does not require specific hardware for time or phase synchronization and thus has been supported by consumer electronics.

For large-scale mass-market indoor localization applications, specific devices or networks may not be affordable. Thus, the existing wireless APs in public areas are used. Additionally, to lower the requirement for the costly database training process, methods such as crowdsourcing [4] and simultaneous localization and mapping (SLAM) [5] have been investigated. Although there are numerous research that has improved the indoor wireless localization performance through advanced models or estimation techniques, the performance of indoor localization has been limited by the issues inherent to wireless measurements. Such issues include the requirement for signal geometry and the existence of RSS fluctuations and disturbances. Due to the complexity of indoor environments and the signal of opportunity (SoO) characteristic of wireless signals, it is difficult to assure wireless localization performance anywhere.

Therefore, two methods are used in this research to enhance indoor wireless localization:

- Method #1: Integration with inertial sensors. The dead-reckoning (DR) solutions from inertial sensors are used to enhance the continuity and robustness of localization.
- Method #2: Setting the wireless localization uncertainty adaptively in data fusion. To achieve this objective, machine learning (ML) methods are used to predict the wireless localization uncertainty, which is further used to set the weight of wireless position updates.

For Method #1, DR is self-contained to provide seamless outdoor/indoor localization solutions [6]. However, low-cost sensors suffer from significant sensor errors. The uncompensated sensor errors may accumulate and cause increasing position errors due to the integral computation in DR algorithm. Therefore, it is vital to correct for DR errors through the integration with external sensors such as wireless localization. In such integration, the DR data is used to build the system model and provide a short-term prediction, while wireless localization solutions are the updates. Kalman filter and particle filter [6] are widely used techniques for information fusion. Profile matching [7] is also used to provide a robuster solution.

For Method #2, there are methods that use adaptive Kalman filters based on variables such as residuals [8] and innovations [9]. These methods are effective in reducing the impact of unreliable wireless measurements when the DR solution is reliable. However, due to the dependency on the DR solution, their performance may be degraded when both wireless and DR data are not accurate. Therefore, one objective of this paper is to estimate wireless localization uncertainty without the need for DR solutions. The research [10] predicts the fingerprinting-based localization uncertainty from three levels (i.e., the signal, geometry, and database levels). In contrast, this paper predicted the localization uncertainty through the use of ML. Two ML methods are used, including the artificial neural network (ANN)-based method and the Gaussian distribution (GD) based method.

ANN has been introduced into the localization area over a decade ago [11] but has not been widely adopted until recent years. Table 1 illustrates part of papers that use ANN in localization within the year of 2018. The majority of works use wireless measurements as the input, while some use data from camera [12], LiDAR [13], inertial [14], and sound [15] sensors. For wireless measurements, RSS (e.g., RSS from WiFi [16], BLE [17], ZigBee [18], RFID [19], cellular [20], and photodiode [21]), RSS features (e.g., two-dimensional RSS map [22], differential RSS [23], and RSS statistics [24]), channel information (e.g., the channel state information (CSI) [25] and channel impulse response (CIR) [26]), and angle-of-arrival (AoA) [27] have been used. These measurements are used for various purposes (i.e., outputs) through ANN. The majority of works directly output two-dimensional or three dimensional locations, while the other works also output information such as attitude angles [28], floor identifications [24], room identifications [16], region identifications [15], AoA [29], distances [18], step lengths [14], and moving status [30]. Additionally, data quality and status indexes such as non-line-of-sight (NLoS) [25], similarity of fingerprints [31] and images [32], localization errors [13] and localization success rate [33] may be generated from ANN.

When investigated on the types of ANN used, CNN [21] is the most widely used. Meanwhile, other types such as RNN [25], RBF [23], and MLP [20] have been used in multiple works. There are also ANN types or algorithms such as GAN [36], CPN [39], ANFIS [18], GCC [29], and TDNN [44]. The majority of works use one to three hidden layers.

Besides ANN, there are ML methods that have been used for indoor fingerprinting. These approaches include nearest neighbors [46], GD-based methods [47], Gauss Process model [48], random forest [49], and support vector machine [50]. When using such methods for wireless fingerprinting, RSS measurements are used as inputs while two-dimensional locations are the outputs. The principle for wireless fingerprinting is to evaluate the similarity between the measured RSS vector and those stored in the database by calculating the likelihood for each RP. As one of the most widely used methods, the GD-based method is selected as the second tool to predict wireless fingerprinting uncertainty in this paper.

Compared to the existing wireless fingerprinting works, the main contributions of this paper are

- Although wireless fingerprinting has been widely used for indoor localization, its performance is difficult to quantify. Thus, this paper predicts fingerprinting-based location uncertainty by given RSS measurements. Two ML methods, including an ANN-based method and the GD-based method, are applied.
- Compared to the existing ML works, this paper uses ML from a new perspective. Specifically, instead of directly estimating the location or navigation states, this paper uses ML to learn and predict the relation between RSS and localization uncertainty. The ML-predicted location uncertainty is further used to set the measurement noises in the dead-reckoning/wireless fingerprinting integrated localization extended Kalman filter (EKF).

This paper is organized as follows. Section 2 illustrates the methodology, including wireless fingerprinting, GD, ANN, and DR/wireless integrated localization. Section 3 describes the tests and results, and Section 4 draws the conclusions.

## 2. Methodology

**Figure 1:**
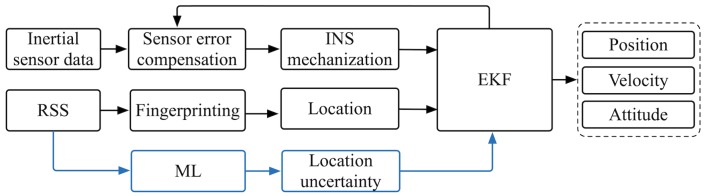
Algorithm flow chart.

As shown in Figure 1, an EKF is used to fuse the inertial sensor data and RSS. Data from inertial sensors is used to predict the states through the INS mechanization and construct the EKF system model, while wireless fingerprinting provides the measurement models. Compared to traditional DR/Wireless fingerprinting integration methods, this paper introduces the fingerprinting uncertainty prediction module, as illustrated by the blue boxes in Figure 1. The fingerprinting uncertainty prediction module is based on ML, which inputs RSS and outputs the predicted fingerprinting-based location uncertainty. The methods of wireless fingerprinting and GD, ANN, and DR/Wireless fingerprinting integrated EKF are described in Section 2.1, Section 2.2 and Section 2.3, respectively.

### 2.1. Wireless Fingerprinting

Fingerprinting consists of the training and localization steps. The training step is conducted to generate a [location, RSS] database that consists of a set of reference points (RPs) with known coordinates and RSS from available APs, while the localization step finds the closest match between the measured RSS and those stored in the database. The reference fingerprint in the database can be written as

(1) $$\lambda_{i}=\left[p_{i},\;\left(\xi_{i,1},r_{i,1}\right),\;\left(\xi_{i,2},r_{i,2}\right),\;\ldots ,\;\left(\xi_{i,j},r_{i,j}\right),\;\ldots ,\;\left(\xi_{i,n_{i}},r_{i,n_{i}}\right)\right]$$

where $\lambda_{i}$ is the reference fingerprint at the *i*th RP in the database and $p_{i}$ is the coordinate of RP *i*. $\xi_{i,j}$ and $r_{i,j}$ are respectively the index (e.g., media access control address) and the RSS with AP *j* at RP *i*. $n_{i}$ is the number of available APs at RP *i*.

The objective for localization is to find the reference fingerprint λ that maximizes β(λ|x) by

(2) $${\hat{\lambda }}_{MLE}=\underset{\lambda }{\arg\max}\left(\beta (\lambda |l)\right)$$

where l is the measured RSS vector. The signs β(·) and argmax represent the probability density function and the arguments of the maxima, respectively. β(λ|l) is the conditional probability of λ on l.

Based on the Bayes’ rule, the probability for the *i*th RP ($\beta (\lambda_{i}|l)$) can be modeled by

(3) $$\beta (\lambda_{i}|l)=\frac{\beta \left(l|\lambda_{i}\right)\beta \left(\lambda_{i}\right)}{\beta (l)}$$

where $\beta (l|\lambda_{i})$ is the likelihood, β(λ) is the prior and β(l) is a normalizing constant. A uniform β(l) is used.

Denote $l=[l_{1}\;\cdots \;l_{j}\;\cdots \;l_{n}]$, where $l_{j}$ is the measured RSS with AP *j*, and *n* is the number of APs in the RSS vector. Assume RSS from the APs are independent with one another, $\beta (l|f_{i})$ can be simplified to

(4) $$\beta \left(l|\lambda_{i}\right)=\beta \left(l_{1}\;\cdots \;l_{j}\;\cdots \;l_{n}|\lambda_{i}\right)=\prod_{j=1}^{n}\beta (l_{j}|r_{i,j})$$

The likelihood $\beta (l_{j}|r_{i,j})$ may be computed by several approaches, such as the histogram [51], GD [47], and log-normal [52] based methods. When the GD-based method is used, $\beta (l_{j}|r_{i,j})$ is modeled as

(5) $$\beta \left(l_{j}|r_{i,j}\right)=\frac{1}{\sigma_{i,j}\sqrt{2\pi }}\exp\left(-\frac{{\left(l_{j}-\mu_{i,j}\right)}^{2}}{2\sigma_{i,j}^{2}}\right)$$

where $\mu_{i,j}$ and $\sigma_{i,j}^{2}$ are the mean and variance of RSS with AP *j* at RP *i*. These values are calculated from static RSS data at the training step.

When using the GD-based method to predict location uncertainty, a [location, RSS] database was additionally trained. Afterwards, the likelihood for each RP was computed by using Equation (3). The κ RPs that had the highest likelihood values are selected. The κ reference RSS vectors at the selected RPs were then fed into the [RSS, location uncertainty] database to obtain the corresponding location uncertainty values. Finally, the predicted location uncertainty was computed as

(6) $$\hat{\epsilon }=\sum_{i=1}^{\kappa }\frac{\epsilon_{i}\beta_{i}}{\sum_{j=1}^{\kappa }\beta_{j}}$$

where $\hat{\epsilon }$ is the predicted position uncertainty value, $\epsilon_{i}$ is the location uncertainty for the *i*th selected RP and $\beta_{i}$ is its likelihood.

### 2.2. Artificial Neural Network

**Figure 2:**
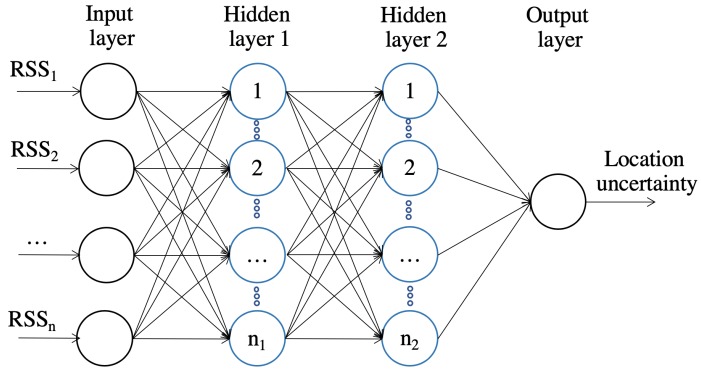
ANN structure.

ANN is actually the framework for using ML algorithms to process complex data inputs. ANN consists of an input layer, an output layer, and at least one hidden layer. Each layer consists of at least one neuron. In this paper, two hidden layers are used, as demonstrated in Figure 2. The neurons are connected via weights that are considered the bulk of the trained neural network in order to estimate the desired output.

This paper applies the multi-layer perceptron (MLP) [53], which is a supervised learning method by using an error back-propagation algorithm. The back-propagation algorithm optimizes the parameters by minimizing the sum of squared errors (i.e., the cost function *c*) of the neurons in the output layer as

(7) $$c=\frac{1}{2m}\sum_{i=1}^{m}\left|\right|\tilde{y}\left(x_{i}\right)-{\hat{y}}^{\iota }\left(x_{i}\right){\left|\right|}^{2}$$

where $x_{i}$ represents the *i*th training example and $\tilde{y}\left(x_{i}\right)$ is the corresponding desired output; $\hat{y}\left(x_{i}\right)$ is the output from the ANN when $x_{i}$ is input. ι denotes the number of layers in the ANN and *m* is the number of training examples.

An important factor in back-propagation is to find the optimum values for weights and biases of the ANN in order to achieve desirable outputs from given inputs. The weights and biases are updated using the four standard back-propagation equations in [54]. These equations calculate the error at the output layer according to (7) and then back-propagate this error to adjust the weights and biases based on a learning rate. Specifically, the back-propagation algorithm can be divided into five steps: input, feed-forward, output error computation, error back-propagation, and output. Refer to [55] for more details about the back-propagation algorithm.

### 2.3. Wireless/Dead-Reckoning Integrated Localization

As shown in Figure 1, an EKF is used to fuse the data from DR and wireless fingerprinting. The simplified psi-angle motion model in [56] is applied as the continuous system model as

(8) $$\dot{x}=Fx+w$$

(9) $$x={\left[\begin{array}{ccccc} \delta p^{n} & \delta v^{n} & \psi & b_{g} & b_{a} \end{array}\right]}^{T}$$

(10) $$F=\left[\begin{array}{ccccc} -[\omega_{en}^{n}\times ] & I_{3\times 3} & 0_{3\times 3} & 0_{3\times 3} & 0_{3\times 3}\\ 0_{3\times 3} & -[\left(2\omega_{ie}^{n}+\omega_{en}^{n}\right)\times ] & \left[f^{n}\times \right] & 0_{3\times 3} & C_{b}^{n}\\ 0_{3\times 3} & 0_{3\times 3} & -[\left(\omega_{ie}^{n}+\omega_{en}^{n}\right)\times ] & -C_{b}^{n} & 0_{3\times 3}\\ 0_{3\times 3} & 0_{3\times 3} & 0_{3\times 3} & -\left(\frac{1}{\tau_{bg}}\right)I_{3\times 3} & 0_{3\times 3}\\ 0_{3\times 3} & 0_{3\times 3} & 0_{3\times 3} & 0_{3\times 3} & -\left(\frac{1}{\tau_{ba}}\right)I_{3\times 3} \end{array}\right]$$

(11) $$w={\left[\begin{array}{ccccc} 0_{3\times 3} & C_{b}^{n}n_{a} & -C_{b}^{n}n_{g} & n_{bg} & n_{ba} \end{array}\right]}^{T}$$

where x, F, and w are the state vector, the dynamics matrix, and the system noise vector, respectively. The states $\delta p^{n}$, $\delta v^{n}$, ψ, $b_{g}$, and $b_{a}$ are the vectors of position errors, velocity errors, attitude errors, gyro biases, and accelerometer biases, respectively; $C_{b}^{n}$ is the direction cosine matrix, which is from the device body frame (i.e., *b*-frame) to the local level frame (i.e., *n*-frame), predicted by the inertial navigation system (INS) mechanization [56]; $f^{n}$ is the specific force vector projected to the *n*-frame, and $\omega_{ie}^{n}$ and $\omega_{en}^{n}$ are the angular rate of the Earth and that of the *n*-frame with respect to the Earth frame (i.e., e-frame), respectively; $n_{g}$ and $n_{a}$ are noises in gyro and accelerometer readings, respectively; $\tau_{bg}$ and $\tau_{ba}$ denote for the correlation time of sensor biases; and $n_{bg}$ and $n_{ba}$ are the driving noises for $b_{g}$ and $b_{a}$. The sign [v×] denotes the skew-symmetric matrix of v. $0_{3\times 3}$ and $I_{3\times 3}$ represent the three-dimensional zero matrix and identify matrix, respectively.

To mitigate DR errors, the pedestrian velocity model [57] is used as a velocity constraint. The corresponding measurement model is

(12) $$\hat{v}-\tilde{v}=H_{v}x+\zeta_{v}$$

(13) $$H_{v}=\left[\begin{array}{ccccc} 0_{3\times 3} & {\left(C_{b}^{n}\right)}^{T} & -{\left(C_{b}^{n}\right)}^{T}\left[v^{n}\times \right] & 0_{3\times 3} & 0_{3\times 3} \end{array}\right]$$

where ${\hat{v}}^{n}$ and ${\tilde{v}}^{n}$ denote the velocity predicted by the system model and that obtained from the pedestrian motion, respectively; $\zeta_{v}$ is the velocity measurement noise vector. The term ${\tilde{v}}^{n}$ can be computed as

(14) $$\tilde{v}={\left[\frac{s_{k}}{t_{k}-t_{k-1}}\;0\;0\right]}^{T}$$

where $s_{k}$ is the step length between time epochs $t_{k-1}$ and $t_{k}$. Refer to [58,59] for details about step detection and step length estimation, respectively.

When Wireless fingerprinting position solutions are available, they are used as EKF location updates. The measurement model is

(15) $${\hat{p}}^{n}-{\tilde{p}}^{n}=H_{p}x+\zeta_{p}$$

(16) $$H_{p}=\left[\begin{array}{ccccc} I_{3\times 3} & 0_{3\times 3} & 0_{3\times 3} & 0_{3\times 3} & 0_{3\times 3} \end{array}\right]$$

where ${\hat{p}}^{n}$ and ${\tilde{p}}^{n}$ denote the position predicted by the system model and that obtained from wireless localization, respectively; $\zeta_{p}$ is the location measurement noise vector.

With the above system and measurement models, the EKF is applied to estimate the state vector in real time. The EKF works by predicting the process states and then obtaining feedback from noisy measurements. Refer to [56] for the details of EKF. In principle, EKF solutions are the weighted average of predictions and measurements. The weights of the predictions and measurements are reflected by the parameters in the system noise matrix and measurement noise matrix, respectively. Since the EKF system model is constructed by self-contained motion models, elements in the system noise matrix are set according to the stochastic sensor characteristics, which can be obtained from in-lab calibration. In contrast, the elements in the measurement noise matrix may vary according to navigation environment changes. Thus, the ML-predicted location uncertainty is further used to set the measurement noises in the EKF.

## 3. Tests and Results

### 3.1. Test Description

Indoor walking tests were conducted in the MacEwan Student Hall, at the University of Calgary. Five Android smart devices were used, including a Samsung Galaxy S4, a Galaxy S7, a Huawei P10, a Lenovo Phab 2 Pro smartphone, and a Nexus 9 tablet.All devices were equipped with three-axis inertial sensors and a WiFi receiver. The data rates were 20 Hz for for inertial sensors and 0.5 Hz for WiFi.

**Figure 3:**
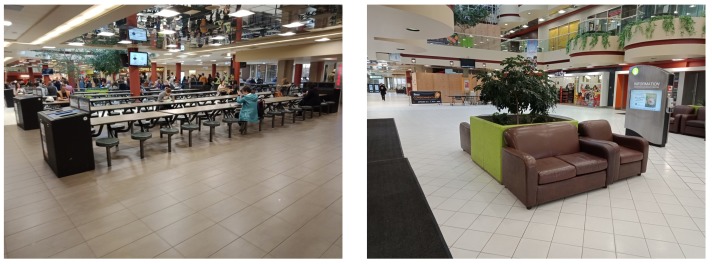
Test environment.

**Figure 4:**
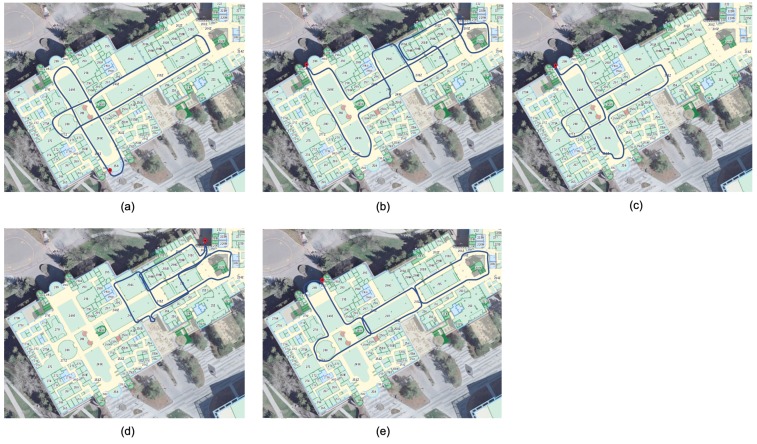
Training trajectories.

**Figure 5:**
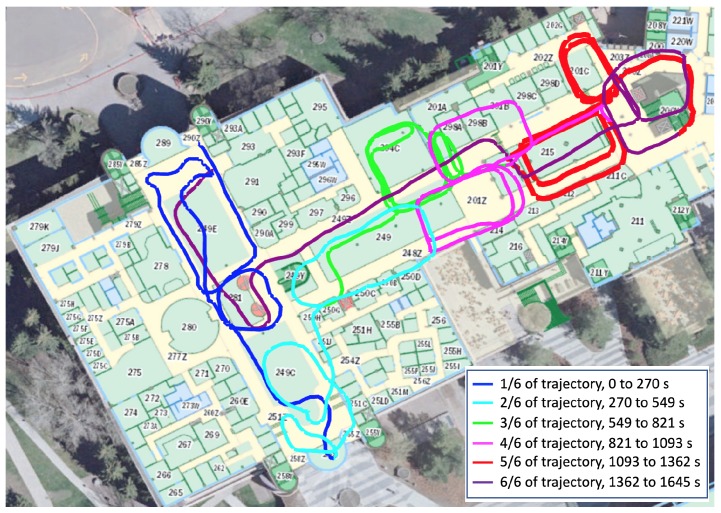
One test trajectory.

The test area was an indoor shopping mall environment, which had a size of approximately 160 m by 90 m. Figure 3 demonstrate the pictures in the test area. To train the wireless fingerprinting database, the walk-survey method was adopted. Two testers participated in the training data collection. Each tester held the five smartphones together and walked within the test area for five training trajectories, as illustrated in Figure 4. There were in total 12,867 WiFi fingerprints in the training data. Each WiFi fingerprint consisted of multiple available MAC address and RSS measurements. In the testing process, each tester held the smartphones horizontally and walked along two pre-designed testing trajectories. Each testing trajectory lasted for around 30 min. One test trajectory is shown in Figure 5, in which various colors represent different data segments with starting and ending time indicated in the legend. There were in total 7692 WiFi fingerprints in the testing data. All the training and testing trajectories started and ended at the same points (as demonstrated by the red dots in Figure 3), so as to provide a loop-closure for the whole trajectory and thus ensure the accuracy for the reference trajectory.

During both the training and testing periods, a Lenovo Phab 2 Pro smartphone was carried by each tester to collect red-green-blue-depth (RGB-D) images and compute SLAM solutions, so as to generate the reference trajectories. Refer to [60] for details about RGB-D-based SLAM. For all the SLAM-based reference solutions, a loop-closure had been detected between the start and end points. The SLAM-based reference location solutions were outputted with a data rate of 1 Hz. When a WiFi data epoch was obtained, the SLAM location solution that had the closest timestamp was used as the reference location.

**Figure 6:**
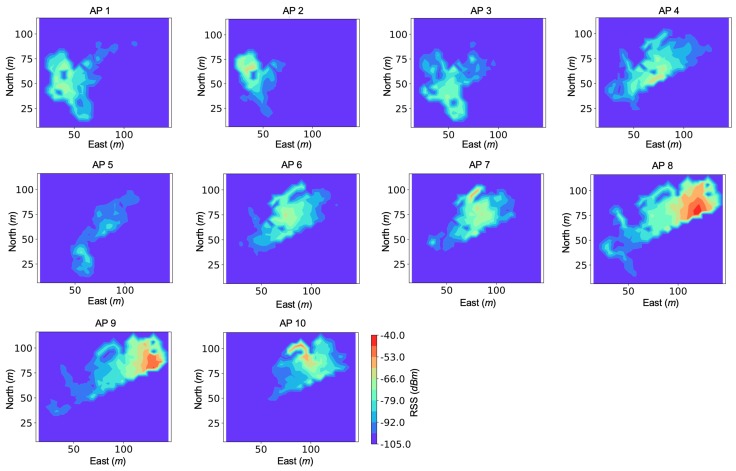
WiFi RSS distributions within test area.

Figure 6 shows the spatial distributions of RSS from the 10 APs used for localization. The AP-localization method in [61] can be used to determine the AP locations. The RSS heatmaps were generated by combining the reference locations from SLAM and the corresponding RSS values that had a similar timestamp. The colors from blue and red represent weak and strong RSS values, respectively. The average coverage area was over 1000 m${}^{2}$ per AP, which was larger than the majority of the existing indoor localization works (e.g., approximately 60 m${}^{2}$ per AP in [35] and 40 m${}^{2}$ per AP in [38]. For practical engineering practice, an AP density of 100 to 400 m${}^{2}$ per AP is commonly used for meter-level WiFi or BLE based localization).

**Figure 7:**
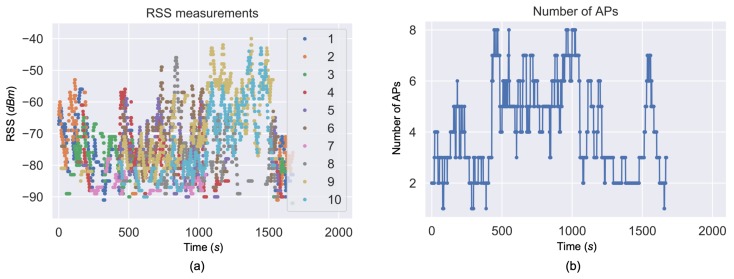
RSS time series (**a**) and number of available APs (**b**).

Figure 7a shows the changes of RSS when the tester walked along the path in Figure 3, while Figure 7b illustrates the number of APs that had a RSS stronger than the threshold −95 dBm. The RSS values changed significantly when the user moved in the test area. During the time period 1200 to 1500 s (i.e., when the tester moved to the north-east corner), RSS from only two or three AP were received; however, the RSS values were strong (e.g., larger than −65 dBm) during this period.

### 3.2. DR and WiFi Fingerprinting Solutions

**Figure 8:**
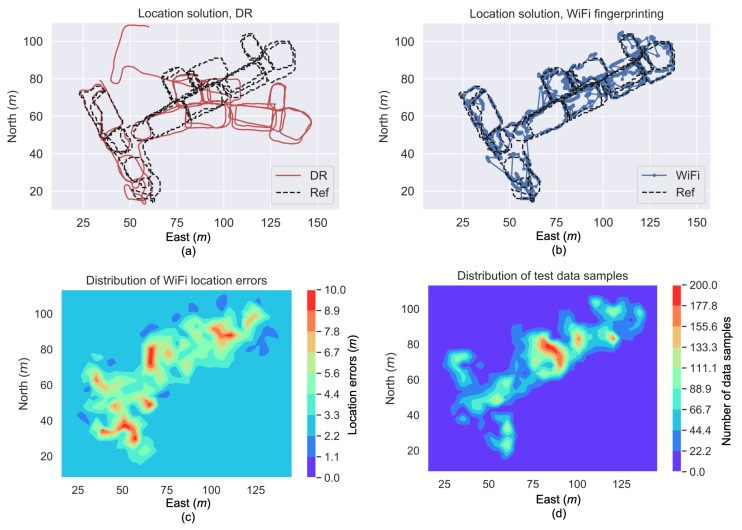
Location solutions from DR (**a**) and WiFi fingerprinting (**b**), heatmap of WiFi fingerprinting location errors (**c**), and heatmap of data samples used for computing the location errors (**d**).

Figure 8a,b illustrates one set of location solutions from DR and WiFi fingerprinting, respectively. The DR solution reflected the moving trajectory but drifted over time, while the WiFi fingerprinting solution fitted with the reference trajectory in the long term but suffered from large position errors (e.g., errors larger than 10 m) at several occasions. Thus, it is worthwhile to integrate DR and WiFi fingerprinting data to obtain a long-term accurate solution.

Figure 8c visualizes the spatial distribution of location errors from WiFi fingerprinting, while Figure 8d illustrates the distribution of data samples used for computing the location errors in Figure 8c. It can be seen that the location uncertainty varied from within 2 m to over 17 m in the test area. This phenomenon indicates the necessity for adaptively setting of the wireless localization uncertainties in multi-sensor integration. The algorithm should be intelligent to reduce the weight of unreliable wireless position updates.

### 3.3. ANN Training and Prediction

**Figure 9:**
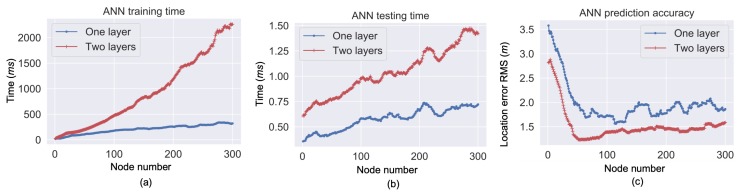
Processing time for ANN training (**a**) and testing (**b**), as well as RMS of differences between ANN-predicted location errors and actual ones (**c**).

As illustrated in Section 2.2, the RSS and wireless localization uncertainty data were combined to train the MLP. The RSS were used as the inputs, while the location uncertainties were used as the outputs. To determine the numbers of layers and neurons in each layer, Figure 9 shows the relationships between node number and training/testing computational time costs and prediction errors by using the experimental data. The computational time was computed in Python 3.6 by using a Macbook that had a 2.5 GHz Intel Core i7 processor. Figure 9 indicates that the MLP structures with two hidden layers generally provided more accurate prediction solutions than those with one hidden layer. Meanwhile, the MLP structures with around 30 neurons in each layer were capable to provide a similar prediction accuracy to those with more neurons. Thus, the MLP structure with two hidden layers, each with 30 neurons, was adopted to balance the computational cost and accuracy.

To train the MLP, the limited-memory Broyden–Fletcher–Goldfarb–Shanno (L-BFGS) algorithm is applied. Refer to [62] for more details of the L-BFGS algorithm. Once the ANN was trained, it was used to predict the wireless fingerprinting uncertainty by given the RSS measurements. The GD method in Section 2.1 was also applied to predict the location uncertainty.

**Figure 10:**
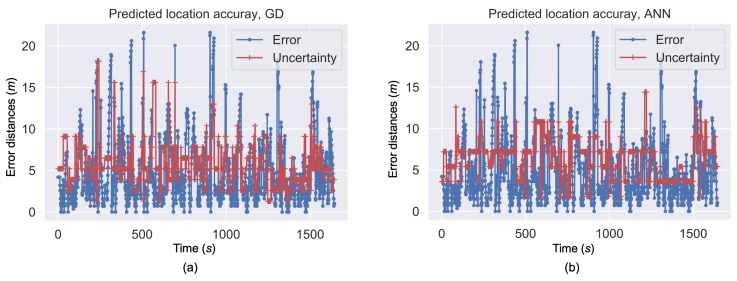
Predicted location uncertainty by GD (**a**) and ANN (**b**).

Figure 10a,b illustrate the time series of the actual fingerprinting-based location errors as well as the location uncertainty that was predicted by the GD and ANN methods, respectively. The prediction solutions from ANN had a lower frequency in variations compared to the GD solutions. This phenomenon is partly because the GD-based method is used on each RP; thus, it has a higher sensitivity than ANN, which is based on parametric models. Generally, the location uncertainty prediction solutions from both approaches generally reflected the varieties of actually location errors. This outcome indicates the feasibility of predicting fingerprinting location uncertainty by using RSS measurements.

**Figure 11:**
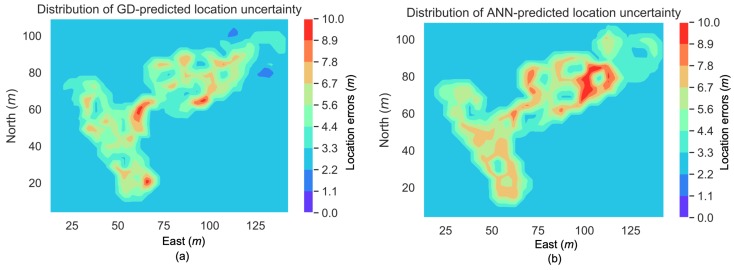
Distribution of location uncertainties predicted by GD (**a**) and ANN (**b**).

Figure 11 illustrates the distributions of the location uncertainties that were predicted by the ANN and GD-based methods, respectively. Compared to Figure 8c, both approaches had reflected the high location errors in regions such as those around the points [80, 110] m (in [east, north]) and [40, 50] m, and the low location errors in regions such as those at around the point [100, 110] m.

### 3.4. Localization with ML-Predicted Location Uncertainty

**Figure 12:**
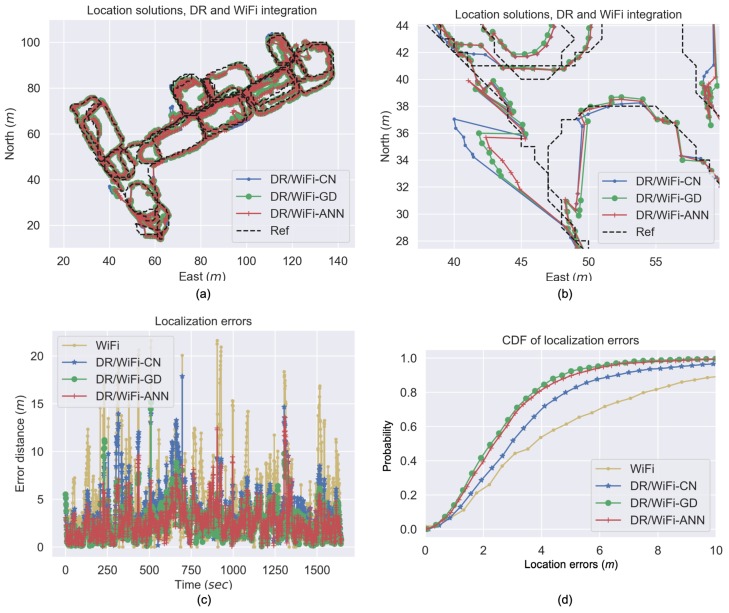
DR/WiFi integrated solutions (**a**), zoomed-in plots (**b**), location errors (**c**) and their CDF (**d**).

The predicted location uncertainties were further used to set the wireless location measurement noises in the EKF. Figure 12a demonstrates the location solutions from DR/WiFi integration that uses three strategies for setting measurement noises. These strategies include DR/WiFi-CN (i.e., setting the measurement noises at a constant value, which was 5 m according to preliminary results), DR/WiFi-GD (i.e., using location uncertainty values predicted by the GD method to set the measurement noises), and DR/WiFi-ANN (i.e., using location uncertainty values predicted by the ANN method to set the measurement noises). Figure 12b is the zoomed-in figures of Figure 12a. It can be seen that the DR/WiFi-CN solution suffered from large position errors at several occasions. A possible reason for such large errors is that the DR/WiFi-CN algorithm was not intelligent enough to increase the measurement noise values when there were large fingerprinting-based location errors. Through the use of the ML-predicted location uncertainties, either the DR/WiFi-GD or DR/WiFi-ANN strategy had reduced these large position errors.

Figure 12c demonstrates the time series of location errors in the test, and Figure 12d illustrates the cumulative distribution function (CDF) of location errors. Table 2 illustrates the corresponding location error statistics, including the standard deviation (STD), mean, root mean squares (RMS) errors, as well as the error within which the probability is 80% (i.e., the 80% error), the error within which the probability is 95% (i.e., the 95% error), and the maximum error. The DR/WiFi-GD and DR/WiFi-ANN strategies provided similar localization performance in general. The DR/WiFi-GD solution had a smaller error RMS, while the DR/WiFi-ANN solution had a smaller maximum error.

Compared to the DR/WiFi-CN strategy, the DR/WiFi-GD and DR/WiFi-ANN strategies had reduced the error RMS values by 27.9% and 23.3%, respectively, and had reduced the 95% errors by 32.3% and 28.7%. Thus, both the DR/WiFi-GD and DR/WiFi-ANN strategies are effective in reducing the location errors for DR/WiFi integration. This phenomenon indicates the effectiveness of adaptively setting location measurement noises in the EKF.

**Figure 13:**
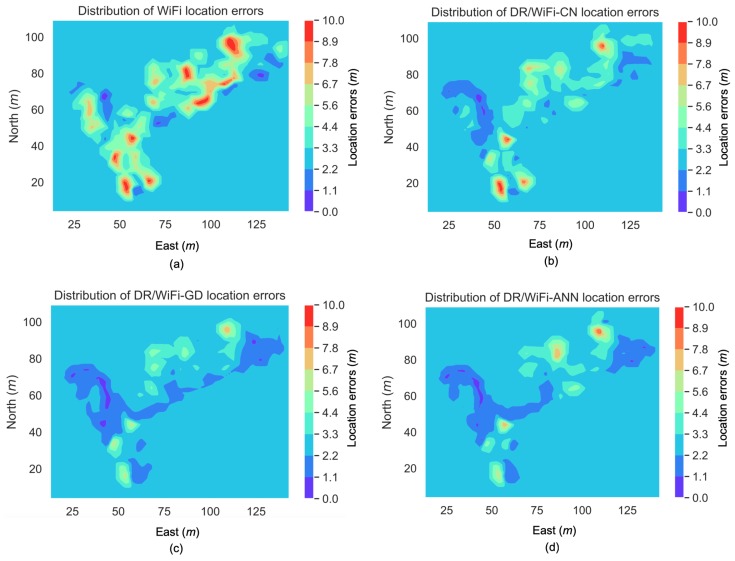
Distribution of location errors from WiFi (**a**), DR/WiFi-CN (**b**), DR/WiFi-GD (**c**), and DR/WiFi-ANN (**d**).

Finally, to visualize the improvement by using DR/WiFi fingerprinting integration and using the ML-predicted location uncertainty in the EKF, Figure 13 shows the distributions of location errors by using WiFi fingerprinting, DR/WiFi-CN, DR/WiFi-GD, and DR/WiFi-ANN. There were several regions that suffered from location errors that were larger than 8 m. When integrated with DR, the majority of these large errors had been mitigated. Furthermore, by introducing the ML-predicted location uncertainty into the EKF, more of such large errors had been mitigated and the majority of location errors were within 4 m.

## 4. Conclusions

This paper has verified the possibility for predicting wireless fingerprinting uncertainty through machine learning. Compared to the traditional DR/WiFi fingerprinting integrated method that uses a constant measurement noise setting for the wireless fingerprinting-based location update, the proposed method, which sets the measurement noise adaptively by using machine learning-based approaches, had reduced the indoor localization errors by 23.3 % to 32.3 %. Therefore, it is suggested to investigate not only the location solution, but also the corresponding location uncertainty from fingerprinting, so as to achieve more reliable indoor localization solutions.

## Figures and Tables

**Table 1:** Part of existing works that use ANN to improve localization (within the year of 2018).

Method	Input	Output	ANN Type/Algorithm	Hidden Layer
[34]	RSS, WiFi	Floor index and location	N/A	N/A
[35]	RSS, WiFi	Location	FF	1–3
[16]	RSS, WiFi	Room index and location	SCG and RBP	2–4
[36]	RSS, WiFi	Location	GAN	3
[37]	RSS, WiFi	Room index	N/A	3
[38]	RSS, WiFi	Location	N/A	1
[39]	RSS, WiFi	Region index	CPN	2
[17]	RSS, BLE	Location	RBF	1
[18]	RSS, ZigBee	Distance	ANFIS	3
[21]	RSS, photodiode	Cell index	CNN	2
[20]	RSS, cellular	Location	MLP	1
[19]	RSS, RFID	Location	FF	2
[31]	RSS	Fingerprint similarity	N/A	1
[40]	RSS	Location	N/A	1
[22]	RSS map	Room index and location	CNN	8
[41]	RSS map	Location	CNN	3
[23]	Differential RSS	Location	RBF	1
[24]	RSS statistics	Floor index	MLP	1
[42]	CSI, WiFi	Location	GCC	N/A
[25]	CSI, WiFi	NLoS identification	RNN	10
[43]	CIR	Location	CNN	3
[26]	CIR, UWB	NLoS identification	CNN	6
[27]	AoA	Location	CNN	8
[29]	GCC	AoA	GCC	2
[15]	Sound	Region index	CNN	10
[44]	Sound	AoA	TDNN	3
[13]	Laser data	Location error	RBF	1
[32]	RGB image	Image similarity	CNN	5
[12]	RGB image	Relation between images	CNN	2
[45]	RGB image	pose	CNN	8
[28]	RGB image	pose	CNN	3
[33]	RGB image, likelihood model, BM model	Localization success rate	CNN	9
[14]	Inertial sensor data	step length	N/A	2–4
[30]	Inertial sensor data	static detection	RNN	4

ANN types or algorithms: CNN-convolution neural network; FF-feed-forward neural network; RBF-radial basis function neural network; BP-back-propagation neural network; GAN-generative adversarial neural network; RNN-recurrent neural network; MLP-multi-layer perceptron; SCG-scaled conjugate gradient; RBP-resilient back propagation; CPN-counter-propagation neural network; ANFIS-adaptive neural fuzzy inference system; GCC-generalized cross-correlation; TDNN-time delay neural network; N/A-not provided.

**Table 2:** Statistics of location errors (unit: m).

Strategy	STD	Mean	RMS	80%	95%	Max
WiFi	3.4	4.9	6.4	7.5	13.7	21.6
DR/WiFi-CN	2.5	3.5	4.3	4.8	8.7	17.9
DR/WiFi-GD	1.7	2.6	3.1	3.7	5.9	15.2
DR/WiFi-ANN	1.9	2.7	3.3	3.9	6.2	13.6

## Abbreviations

The following abbreviations are used in this manuscript:

ANFIS	adaptive neural fuzzy inference system
ANN	artificial neural network
AoA	angle-of-arrival
AP	access point
BDS	BeiDou navigation satellite system
BLE	bluetooth low energy
BP	back-propagation
CNN	convolution neural network
CPN	counter propagation neural network
DOP	dilution of precision
DR	dead-reckoning
EKF	extended Kalman filter
FF	feed-forward neural network
GAN	generative adversarial neural network
GCC	generalized cross correlation
GD	gaussian distribution
GNSS	global navigation satellite systems
GPS	global positioning services
INS	inertial navigation system
L-BFGS	limited-memory Broyden–Fletcher–Goldfarb–Shanno
LPWAN	low-power wide-area network
ML	machine learning
MLP	multi-layer perceptron
N/A	not provided
NLoS	non-line-of-sight
RBF	radial basis function neural network
RBP	resilient back propagation
RFID	radio frequency identification
RGB-D	red-green-blue-depth
RMS	root mean squares
RNN	recurrent neural network
RP	reference point
RSS	received signal strength
SCG	scaled conjugate gradient
SLAM	simultaneous localization and mapping
SoO	signal of opportunity
STD	standard deviation
TDNN	time delay neural network
ToA	time-of-arrival
UTC	coordinated universal time
UWB	ultra-wide-band
WiFi	wireless local area network
